# Fear avoidance beliefs, anxiety, and depression in healthy individuals and persons with vestibular disorders across cultures

**DOI:** 10.3389/fneur.2023.1296411

**Published:** 2023-12-01

**Authors:** Lien Van Laer, Pamela M. Dunlap, Luc Vereeck, Erwin Hendriks, Morgana Sluydts, Susan L. Whitney

**Affiliations:** ^1^Department of Rehabilitation Sciences and Physiotherapy/Movant, Faculty of Medicine and Health Sciences, University of Antwerp, Antwerp, Belgium; ^2^Department of Physical Therapy, University of Pittsburgh, Pittsburgh, PA, United States; ^3^Unit of Physiotherapy, Organizational Part of the Orthopedics Department, Erasmus Medical Centre, Rotterdam, Netherlands; ^4^European Institute for Otorhinolaryngology—Head and Neck Surgery (ORL-HNS), Sint-Augustinus Hospital Antwerp, Antwerp, Belgium

**Keywords:** vestibular disorders, fear avoidance beliefs, anxiety, depression, dizziness, balance confidence

## Abstract

**Background/introduction:**

In persons with vestibular disorders, disturbed vestibular input and accompanying dizziness can be associated with anxiety or depression. To avoid dizziness, persons with vestibular disorders can develop mal-adaptive fear avoidance behaviors which can negatively influence daily life functioning. The aims of this study were to (1) document different psychological factors in patients with vestibular disorders and healthy adults across cultures and (2) to assess the convergent validity of the 9-item Vestibular Activities Avoidance Instrument (VAAI), which quantifies fear avoidance beliefs.

**Methods:**

Psychological factors and disability were measured in Dutch-speaking healthy adults and English- and Dutch-speaking persons with vestibular disorders using the VAAI, the Dizziness Handicap Inventory (DHI), the Hospital Anxiety and Depression Scale (HADS) and the Activities-Specific Balance Confidence Scale (ABC). The convergent validity of the VAAI was investigated by performing correlation analyses between the VAAI, the DHI, the HADS, and the ABC.

**Results:**

A total of 151 Dutch-speaking healthy adults, 404 English-speaking participants with vestibular disorders, and 126 Dutch-speaking participants with vestibular disorders were included. Participants with vestibular disorders presented with higher levels of fear avoidance beliefs (VAAI), perceived disability (DHI), anxiety and depression (HADS), and lower confidence during balance activities (ABC) compared to healthy adults. Regarding the convergent validity of the VAAI, there were moderate to strong correlation coefficients (*r* = 0.39–0.74) between fear avoidance and the DHI, HADS, and ABC in participants with vestibular disorders.

**Conclusions:**

Participants with vestibular disorders report a higher psychological burden compared to healthy adults. These results emphasize the importance of assessing psychological factors in persons with vestibular disorders. In addition, evidence was provided for convergent validity, supporting the VAAI as a valid outcome measure across cultures.

## 1 Introduction

With a prevalence ranging between 2.8 and 6.5% across cultures, vestibular disorders are common across the world (1, 2). Dizziness is a well-known symptom of vestibular disorders and can have a detrimental impact on functioning during daily life (3). Moreover, dizziness often leads to frequent consultations with healthcare providers (4). Dizziness and its impact can be quantified by the Dizziness Handicap Inventory (DHI) (5). The DHI not only covers the physical and functional aspects of dizziness but the emotional aspect as well, suggesting that there is an association between dizziness and emotional/psychological factors. Persons with vestibular disorders and higher levels of anxiety and depression experience greater dizziness (6–9). The association between dizziness and psychological factors was supported by imaging and animal studies unraveling connections between the vestibular system and brain areas involving cognition and emotional processes (10). Furthermore, Hilber developed an internal-fake-news-model in which the interaction between both—dizziness and psychological factors- was clarified. In Hilber's model, the continuous disturbed vestibular input leads to chronic stress, anxiety and depression (10). In addition to the association between dizziness and psychological factors, anxiety and depression are more common in vestibular patients compared to healthy controls (11–17). Although several types of vestibular disorders exist, there is no consensus whether the severity of psychological factors differs between vestibular disorders. A recent study in which anxiety and depression were compared between persons with Persistent Postural Perceptual Dizziness (PPPD) and persons with dizziness, found no significant differences in median anxiety or depression scores between groups. On the other hand, a significantly higher number of persons with pathological anxiety was observed in the PPPD group (18). Another study with a larger population also found higher anxiety levels in PPPD patients compared to other vestibular disorders (19). Various questionnaires are available to record anxiety or depression such as the Hospital Anxiety and Depression Scale (HADS) (20) or the Generalized Anxiety Disorder (GAD)-7 (21). Despite demonstrating good to excellent psychometric qualities and widespread utilization in research (21–23), these questionnaires are not frequently incorporated into the standard assessment of patients with vestibular disorders in clinical practice (24). However, without examining psychological factors, relevant treatment options such as Cognitive Behavioral Therapy (CBT) might be neglected (25, 26).

Another interesting psychological factor, namely fear avoidance, seems worthy of consideration among persons with vestibular disorders. Fear avoidance is a behavioral response to avoid provocation of dizziness (27). In vestibular disorders, dizziness and/or imbalance can be triggered by head movements or visual stimuli. Although exposure to these triggers is needed to decrease motion and visual sensitivity (28), a common behavioral response is to avoid symptoms being triggered. Hence, fear avoidance beliefs might develop in this population and thereby compromising recovery.

Fear avoidance beliefs can be assessed by the Vestibular Activities Avoidance Instrument (VAAI) (27). This short questionnaire, consisting of nine items, has excellent internal consistency and reliability (27, 29). Although anxiety and depression can develop as a response to the perceived consequences of ongoing disruptions in vestibular input (10), fear avoidance beliefs might rather be seen as an anticipatory or proactive step to avoid the disturbed vestibular input (10, 27). Therefore, both types of psychological factors (anxiety and depression vs. fear avoidance beliefs) seem relevant to assess but are different constructs. A third psychological factor to consider is balance confidence. The Activities-Specific Balance Confidence scale (ABC) provides data about how confident a patient feels when performing balance activities such as going up and down the stairs (30). This questionnaire indicates the level of self-confidence while doing balance activities and has been associated with actual balance performance in persons with vestibular disorders (31).

To summarize, the emotional component of the DHI, HADS, VAAI, and ABC assess different psychological factors. The VAAI has only recently been developed and research on its validity is indicated. The goal of this study is to (1) document the different psychological factors as measured by the VAAI, DHI, HADS, and ABC in persons with vestibular disorders and healthy adults across cultures and (2) to assess the convergent validity of the VAAI.

## 2 Materials and methods

### 2.1 Participants

#### 2.1.1 Dutch-speaking healthy participants

A convenience sample of healthy adults over the age of 18 were recruited from the vicinity of Antwerp, through advertisements and personal contacts of the researchers, students and staff within the Department of Rehabilitation Sciences and Physical Therapy, University of Antwerp, Belgium. Participants were eligible to participate if they were without complaints of dizziness in the past 6 months. A total of 154 participants volunteered but three were excluded because of a Ramsey Hunt recurrence < 3 months prior to the study with hearing loss and unilateral peripheral vestibular hypofunction, one participant was attending physical therapy sessions for balance problems at the time, and one participant had bilateral knee prostheses impeding balance performance. Therefore, the sample of healthy participants included 151 adults. The study was approved by the University of Antwerp Ethics Committee (reference number: 18/12/162) and all participants provided informed consent.

#### 2.1.2 Dutch-speaking participants with vestibular disorders

A convenience sample was recruited through the ENT department of three hospitals, namely the Erasmus Medical Centre in Rotterdam, the Netherlands, the Augustinus Hospital in Antwerp (European Institute for Otorhinolaryngology—Head and Neck Surgery), Belgium and the Antwerp University Hospital, Antwerp, Belgium. The recruitment period ran from January 1, 2019, to August 31, 2020, for the first two centers and from December 2, 2021, to July 7, 2022 for the Antwerp University Hospital. Participants older than 18 years visiting one of the three centers for consultation were asked to participate in the study. A medical history was taken in all participants and vestibular function testing and imaging were carried out if indicated. This multicenter study was approved by the University of Antwerp Ethics Committee (reference numbers: 18/12/162 and 21/12/181) and the UMC Erasmus Hospital Ethics Committee (reference number: WT/aj/MEC-2018-1190). All participants provided informed consent.

#### 2.1.3 English-speaking participants with vestibular disorders

A convenience sample was recruited from a tertiary care balance disorders clinic and outpatient vestibular rehabilitation clinics. Eligibility criteria included: reported current dizziness and/or imbalance, aged 18–100 years old, and English-speaking. The recruitment took place from February 20, 2018, to December 31, 2019. The study was approved by the University of Pittsburgh Institutional Review Board (19030119) and all participants provided informed consent. Merging of the data was approved based on a data exchange agreement between the University of Pittsburgh (USA), University of Antwerp (Belgium), the Erasmus Medical Centre in Rotterdam (the Netherlands) and the European Institute for ORL-HNS at the Sint-Augustinus Hospital in Antwerp (Belgium).

### 2.2 Outcome measures

Healthy participants (*n* = 151) completed the Dutch versions of the VAAI (29), the Dizziness Handicap Inventory (DHI) (32), the Activity-specific Balance Confidence Scale (ABC) (30), and the Hospital Anxiety and Depression Scale (HADS) (33) with pencil and paper during the study visit. Dutch-speaking participants with vestibular disorders (*n* = 126) completed the Dutch versions of the VAAI, the DHI, the ABC, and the HADS with pencil and paper (Antwerp site) or computerized versions (Rotterdam site) on the same day as their visit to the physician (29, 30, 32, 33). In twenty-five Dutch-speaking participants with vestibular disorders (19.8%) the ABC was not completed. The English-speaking participants with vestibular disorders completed computerized versions of the VAAI and HADS on the same day as their visit with the physician or physical therapist (20, 27, 34). The DHI was only completed by 82% (*n* = 332) of English-speaking participants as it was abstracted from the medical record (5). The ABC was not completed by the English-speaking participants.

#### 2.2.1 Vestibular Activities Avoidance Instrument

The English and Dutch versions of the VAAI have demonstrated excellent internal consistency and test-retest reliability in healthy adults and persons with vestibular disorders (27, 29). The VAAI has recently been shortened to include only nine items to decrease the time burden for individuals completing the questionnaire (27). The 9-item VAAI score ranges from 0 to 54 meaning that each item is scored from 0 to 6: 0 = strongly disagree, 1 = disagree, 2 = somewhat disagree, 3 = neutral, 4 = somewhat agree, 5 = agree and 6 = strongly agree (27). The higher the score, the higher the chance of presence of fear avoidance beliefs. In this analysis, the nine items were abstracted from the 81-item VAAI.

#### 2.2.2 Hospital Anxiety and Depression Scale

The HADS was developed to screen for clinically significant anxiety and depression symptoms (20). The HADS anxiety and depression subscales each range from 0 to 21 with higher scores indicating more severe anxiety and depression. Those with scores > 7 on either subscale are considered having borderline abnormal (8–10) or abnormal levels (>10) of anxiety and/or depressive symptoms (20). The Dutch and English versions of the HADS have demonstrated good psychometric properties (33, 34).

#### 2.2.3 Dizziness Handicap Inventory

The DHI ranges in score from 0 to 100 with higher scores indicating greater perceived handicap due to dizziness and imbalance (5). The Dutch version of the DHI has previously demonstrated excellent test-retest and internal consistency reliability (32, 35). It has been suggested that DHI scores of 0–30 indicate mild handicap impairment, scores ranging from 31 to 60 indicate moderate handicap and scores >60 indicate severe handicap (36). Recently, Graham et al. found that scores of 60 or higher were likely to have a functional or psychiatric disorder with or without a structural neurotological disorder (specificity = 0.88) and scores 30 and lower were likely to have a structural neurotological disorder only (specificity = 0.98) as a cause of their dizziness (37).

#### 2.2.4 Activities-Specific Balance Confidence Scale

The ABC-scale examines the extent to which people are confident they can perform various activities from everyday life without a loss of balance (30). In total, 16 activities are included and are scored from 0 to 100 (0 = no confidence; 100 = maximal confidence). The total ABC score is the sum of the individual item scores, which is averaged to obtain a percentage score. The total score can be categorized from 0–50, 51–80, and 81–100 indicating low-, moderate- and high-level functioning, respectively (38). The Dutch version of the ABC showed moderate correlations with balance performance (31).

### 2.3 Statistical analyses

Descriptive statistics were used to characterize the demographic information and outcome measure scores among the three participant groups. One-way analysis of variance (ANOVA) and Chi-square tests were used to determine differences in demographic characteristics and outcome measure scores between the three groups. For *post-hoc* pairwise comparisons, the Bonferroni procedure was used to correct for a Type II error. The Kruskal-Wallis test was used when the data did not meet the assumptions for a one-way ANOVA. Independent sample *t*-tests were performed to compare the Dutch- and English-speaking participants with a vestibular disorder. Convergent validity was analyzed by examining the relationship between the 9-item VAAI and the DHI, ABC, and HADS subscale scores using Spearman's correlation coefficients. Correlation coefficients were interpreted as follows: <0.3 were considered weak, 0.3–0.5 were considered moderate, and >0.5 were considered strong (39). Furthermore, we used one-way ANOVA or the Kruskal-Wallis test to compare VAAI scores between the HADS, DHI and ABC subgroups. The subgroups were made as follows: normal (0–7/21) or abnormal anxiety and depression subscale scores (8–21/21) on the HADS (20); mild (0–30/100), moderate (31–60/100) or severe perceived handicap (61–100/100) on the DHI (36); low-level (0–50/100), moderate-level (51–80/100) or high-level functioning (81/100) on the ABC (38).

## 3 Results

### 3.1 Baseline characteristics across groups

The mean age (SD) of the Dutch-speaking healthy participants was 50.9 (18.5), the Dutch-speaking participants with vestibular disorders was 57.2 (14.2), and the English-speaking participants with vestibular disorders was 54.0 (17.0) (*p* = 0.008) (Table 1). The only groups that were significantly different in age were the Dutch-speaking healthy participants (50.9 ± 18.5 years) and Dutch-speaking participants with vestibular disorders (57.2 ± 14.2 years) (*p* = 0.006). The gender distribution was similar across all groups. Among the patient groups, the median duration of dizziness symptoms in months was twelve among the Dutch-speaking participants and eight among the English-speaking participants. Diagnostic categories were created based on the ICD-10 diagnosis codes: Benign Paroxysmal Positional Vertigo (BPPV), other peripheral (e.g., uni- or bilateral vestibular hypofunction or Menière's Disease), central vestibular disorders (e.g., vestibular migraine), unspecified vestibular disorders, gait disorders or functional disorders. The distribution of participants with vestibular disorders in each category differed between groups such that the Dutch-speaking participants with vestibular disorders were more likely to be diagnosed with peripheral vestibular disorders while more English-speaking participants were diagnosed with unspecified dizziness.

**Table 1:** Baseline characteristics and comparison across groups.

	**Dutch-speaking**		**English-speaking**	***P*-value**
	**Healthy participants**	**Participants with vestibular disorders**	**Participants with vestibular disorders**	
	***N*** = **151**	***N*** = **126**	***N*** = **404**	
Age in years, mean (standard deviation)	50.9 (18.5)^*^	57.2 (14.2)^*^	54.0 (17.0)	0.008
Female, *n* (%)	90 (59.6)	73 (57.9)	261 (64.6)	0.300
Duration of symptoms, median (IQR)	–	12 (5–36)^***^	8 (3–24)^***^	0.005
Participants with a duration of symptoms < 3 months, *n* (%)	–	15 (12.3)^***^	78 (20.8)^***^	0.044
**Primary diagnosis**, ***n*** **(%)**				< 0.001
BPPV	–	16 (12.7)	22 (5.4)	
Other peripheral	–	80 (63.5)^**^	138 (34.2)^**^	
Central	–	18 (14.3)	78 (19.3)	
Unspecified	–	8 (6.3)^**^	153 (37.9)^**^	
Gait disorder	–	2 (1.6)	13 (3.2)	
Functional disorder	–	2 (1.6)	0 (0)	

IQR = interquartile range; BPPV = benign paroxysmal positional vertigo.

^*^Post-hoc analyses revealed a significant difference in age between the Dutch-speaking healthy participants and Dutch-speaking participants with vestibular disorders (*p* = 0.006).

^**^Post-hoc analyses revealed that the distribution of primary diagnosis-categories other peripheral (*p* < 0.001) and unspecified disorders (*p* < 0.001) significantly differed between the Dutch- and English-speaking patients.

^***^In four Dutch-speaking participants and 29 English-speaking participants, the duration of symptoms was not reported.

As expected, all outcome measure scores were different between the healthy participants and the two patient groups (Table 2). The Dutch-speaking participants with vestibular disorders had significantly higher DHI functional [18.2 (9)], physical [16.5 (6.4)], and total scores [47.2 (20.4)] as well as higher VAAI scores [28.4 (11.8)] compared to the English-speaking participants with vestibular disorders [DHI function = 14.1 (10.3), DHI physical = 12.3 (6.7), DHI total = 37.7 (22.8), VAAI = 25.3 (14)]. Across all participants with vestibular disorders, there was a very weak correlation between duration of symptoms and the HADS-D (ρ = 0.114, *p* = 0.012) and DHI total score (ρ = 0.134, *p* = 0.005) indicating that individuals with a longer symptom duration were more likely to rate higher depressive symptoms and perceived dizziness-related handicap.

**Table 2:** Comparison of outcome measures across healthy participants and the Dutch- and English-speaking participants with vestibular disorders.

	**Dutch-speaking**		**English-speaking**	***P*-value on the overall ANOVA or Kruskal Wallis test**
	**Healthy participants**	**Participants with vestibular disorders**	**Participants with vestibular disorders**	
	***n*** = **151**	***n*** = **126**	***n*** = **404**	
	**Mean (SD);** ***N***	**Mean (SD);** ***N***	**Mean (SD);** ***N***	
Activity-Specific Balance Confidence Scale (0–100)	92.9 (8.4); 151	61.7 (21.6); 101	–	< 0.001
Dizziness Handicap Inventory—Functional^*^	1.3 (3.2); 151	18.2 (9); 123	14.1 (10.3); 332	< 0.001
Dizziness Handicap Inventory—Emotional^∧^	0.7 (2.5); 151	12.7 (8.4); 123	11.3 (8.8); 332	< 0.001
Dizziness Handicap Inventory—Physical^*^	2.1 (3.7); 151	16.5 (6.4); 123	12.3 (6.7); 332	< 0.001
Dizziness Handicap Inventory Total (0–100)^*^	4.2 (8.0); 151	47.2 (20.4); 123	37.7 (22.8); 332	< 0.001
Hospital Anxiety and Depression Scale—Anxiety (0–21)^∧^	4.2 (3.4); 151	6.8 (4.4); 117	7.2 (4.4); 404	< 0.001
Hospital Anxiety and Depression Scale—Depression (0–21)^∧^	2.0 (2.1); 151	6.0 (4.7); 117	5.5 (4.1); 404	< 0.001
Vestibular Activities Avoidance Instrument 9 Item (0–54)^*^	2.4 (5.9); 151	28.4 (11.8); 126	25.3 (14); 404	< 0.001

*n* = the total number of the three groups; *N* = the specific number of participants that filled out that questionnaire; N/A = not applicable.

Healthy control participants were significantly different from Dutch- and English-speaking participants with vestibular disorders for all measures.

^*^Post-hoc analyses revealed that Dutch-speaking participants with vestibular disorders had significantly higher 9-item Vestibular Activities Avoidance Instrument scores (*p* = 0.038), Dizziness Handicap Inventory Function subscale scores (*p* < 0.001), Physical subscale scores (*p* < 0.001), and Total scores (*p* < 0.001) compared to English-speaking participants with vestibular disorders.

^∧^*Post-hoc* analyses revealed that Dutch-speaking participants with vestibular disorders showed no significant differences on the Dizziness Handicap Inventory Emotional subscale (*p* = 0.292), Hospital Anxiety and Depression—Anxiety subscale (*p* = 0.902) or Hospital Anxiety and Depression—Depression subscale (*p* = 1.000) compared to the English-speaking participants with vestibular disorders.

**Figure 1:**
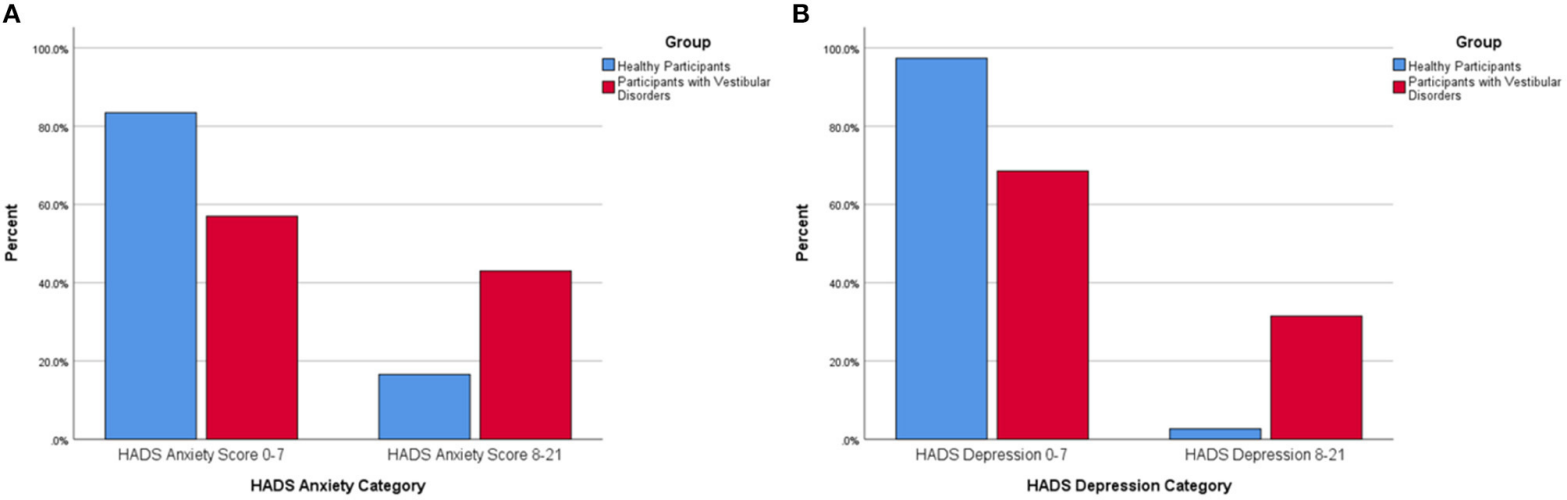
**(A)** Percentage of healthy participants and participants with vestibular disorders reporting abnormal anxiety symptoms via the Hospital Anxiety and Depression-Anxiety (HADS-A) subscore (0–7 equals a normal HADS-A score, 8–21 equals an abnormal HADS-A score). There was a significantly larger percentage of participants with vestibular disorders within the abnormal range on the Hospital Anxiety and Depression-Anxiety Subscale (8–21) compared to healthy adults. **(B)** Percentage of healthy participants and participants with vestibular disorders reporting abnormal depression symptoms via the Hospital Anxiety and Depression-Depression (HADS-D) subscore (0–7 equals a normal HADS-D score, 8–21 equals an abnormal HADS-D score). There was a significantly larger percentage of participants with vestibular disorders within the abnormal range on the Hospital Anxiety and Depression-Depression Subscale (8–21) compared to healthy adults.

Forty-three percent of all participants with vestibular disorders were within the abnormal range on the HADS anxiety subscale compared to 16.6% of the healthy adults (*p* < 0.001) (Figure 1A). Only 2.6% of healthy adults rated abnormally high depressive symptoms on the HADS-D whereas 31.5% of all participants with vestibular disorders were in the abnormal range (*p* < 0.001) (Figure 1B).

### 3.2 Convergent validity of VAAI

The VAAI demonstrated significant associations with all outcome measures (Table 3). The VAAI had a moderate negative relationship with the ABC among Dutch-speaking healthy adults (ρ = −0.42, *p* < 0.001) and participants with a vestibular disorder (ρ = −0.47, *p* < 0.001) indicating that greater fear avoidance beliefs were associated with lower balance confidence. The VAAI had strong positive relationships with the DHI total score among both patient groups (ρ = 0.74, ρ = 0.79, *p* < 0.001) and healthy adults (ρ = 0.62, *p* < 0.001). The VAAI had a weak positive relationship with the HADS anxiety (ρ = 0.29, *p* < 0.001) among healthy adults and a moderate positive relationship with the HADS anxiety among both patient groups (ρ = 0.39, ρ = 0.47, *p* < 0.001). The VAAI had a moderate relationship with the HADS depression subscale among healthy adults (ρ = 0.32, *p* < 0.001) and a strong relationship in both the Dutch- and English-speaking patient groups, respectively (ρ = 0.63, ρ = 0.64, *p* < 0.001) indicating that greater fear avoidance beliefs were associated with greater reported anxiety and depression symptoms.

**Table 3:** Convergent Validity of the VAAI among the Dutch- and English-speaking participants.

	**Dutch-speaking healthy**	**Dutch-speaking vestibular**	**English-speaking vestibular**
	***N*** = **151**	***N*** = **126**	***N*** = **404**
Activity-Specific Balance Confidence Scale	−0.42^*^	−0.47^*^	–
Dizziness Handicap Inventory—Functional	0.47^*^	0.72^*^	0.75^*^
Dizziness Handicap Inventory—Emotional	0.60^*^	0.67^*^	0.68^*^
Dizziness Handicap Inventory—Physical	0.60^*^	0.45^*^	0.62^*^
Dizziness Handicap Inventory Total	0.62^*^	0.74^*^	0.79^*^
Hospital Anxiety and Depression Scale—Anxiety	0.29^*^	0.39^*^	0.47^*^
Hospital Anxiety and Depression Scale—Depression	0.32^*^	0.63^*^	0.64^*^

Spearman's rho correlation coefficients are reported.

^*^p < 0.001.

### 3.3 VAAI scores in HADS, DHI and ABC subgroups

**Figure 2:**
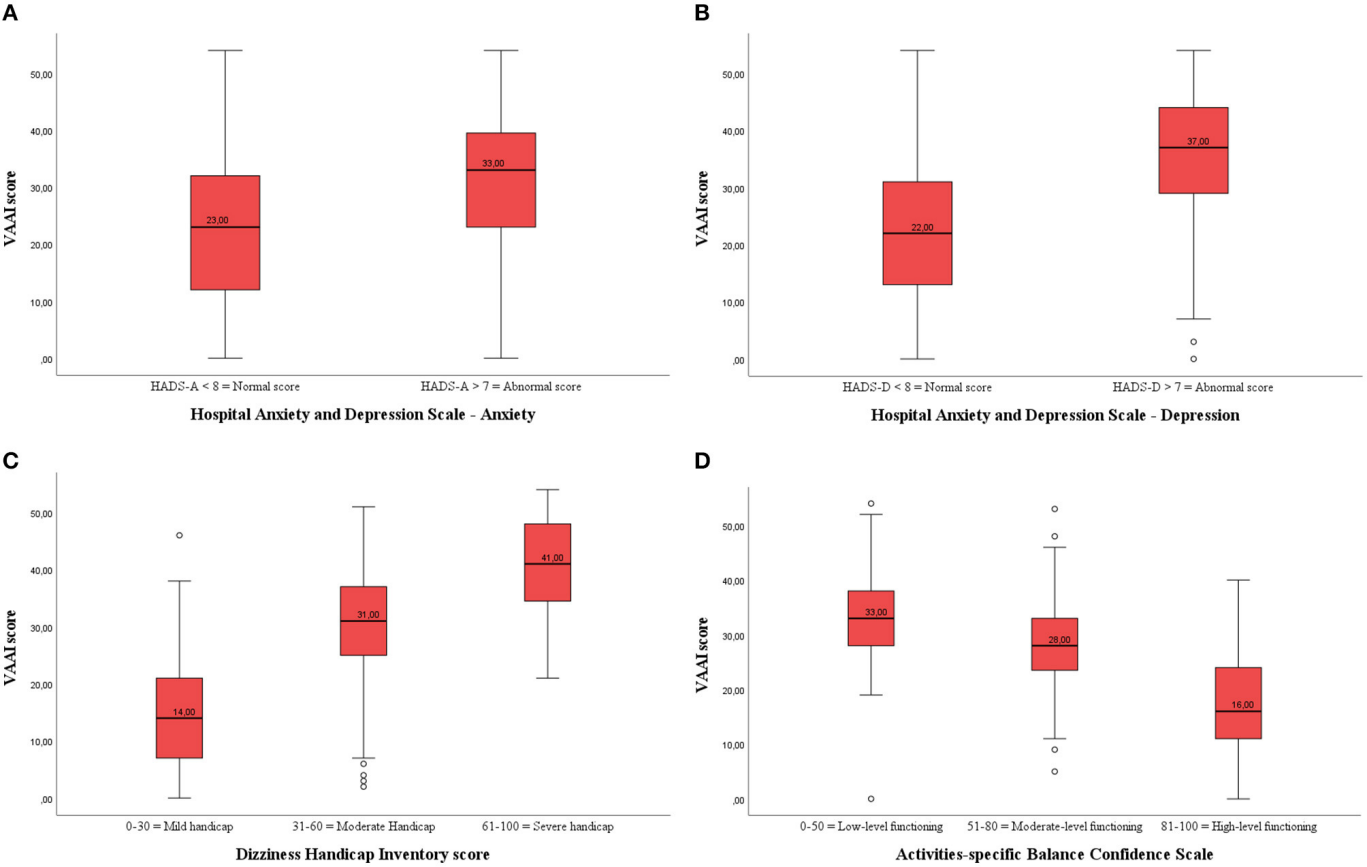
**(A)** Boxplot of the VAAI score per Hospital Anxiety and Depression-Anxiety (HADS-A) subscale in participants with vestibular disorders (both English- and Dutch-speaking). There was a significant difference in VAAI score between participants with vestibular disorders with a normal or abnormal HADS-A score. **(B)** Boxplot of the VAAI score per Hospital Anxiety and Depression-Depression (HADS-D) subscale in participants with vestibular disorders (both English- and Dutch-speaking). There was a significant difference in VAAI score between participants with vestibular disorders with a normal or abnormal HADS-D score. **(C)** Boxplot of the VAAI score per Dizziness Handicap Inventory (DHI) subscale in participants with vestibular disorders (both English- and Dutch-speaking). There was a significant difference in VAAI score between participants with vestibular disorders with a mild, moderate or severe handicap. **(D)** Boxplot of the VAAI score per Activities-Specific Balance Confidence (ABC) subscale in participants with vestibular disorders (both English- and Dutch-speaking). There was a significant difference in VAAI score between participants with vestibular disorders with low-level, moderate-level or high-level functioning.

Both healthy adults and participants with vestibular disorders that scored within the normal range of the HADS anxiety subscale had significantly lower VAAI scores than those who were in the abnormal range on the HADS anxiety subscale (Table 4; Figure 2A). Similarly, healthy adults and participants with vestibular disorders who were within the normal range of the HADS depression subscale had significantly lower VAAI scores (Figure 2B). However, there were only four healthy adults that scored within the abnormal range of the HADS depression subscale.

**Table 4:** Mean VAAI-9 score by outcome measure category.

	**Healthy participants**	**All participants with vestibular disorders**
	**Mean VAAI (SD) (*****n*** = **151)**	**Mean VAAI (SD) (*****n*** = **530)**
**HADS-anxiety**		
0–7	1.7 (4.8)^*^N = 126	22.2 (13)^*^N = 297
>7	6.3 (8.7)^*^N = 25	31.1 (12.7)^*^N = 224
**HADS-depression**		
0–7	2.1 (5.1)^∧^*N* = 147	21.6 (12.4)^*^N = 357
>7	13.5 (16.3)^∧^*N* = 4	35.8 (10.9)^*^N = 164
**Dizziness Handicap Inventory**		
0–30	2.0 (4.2)^*^N = 149	14.8 (9.5)^*^N = 183
31–60	37.5 (0.7)^*^N = 2	30.0 (9.3)^*^N = 180
61–100	–	41.0 (8.5)^*^N = 88
**Activities-Specific**		*N* = 101
**Balance Confidence**		
**Scale**		
0–50	37.0 (N/A)^*^N = 1	33.4 (10.5)^*^N = 32
51–80	9.0 (12.8)^*^N = 9	28.3 (9.8)^*^N = 47
81-100	1.8 (3.9)^*^N = 141	18.0 (11.1)^*^N = 22

^*^p < 0.001; ^∧^*p* = 0.01; N/A, not applicable.

There were two healthy adults who scored from 31 to 60 on the DHI and none scored above 60. Therefore, comparisons in between DHI-subgroups were only made among the participants with vestibular disorders. Those participants who scored 0-30 on the DHI (N = 183) had an average VAAI score of 14.8 (9.5), those who scored 31–60 (*N* = 180) on the DHI had an average VAAI score of 30.0 (9.3), and those who scored >60 on the DHI (*N* = 88) had an average VAAI score of 41.0 (8.5) (*p* < 0.001) (Figure 2C). *Post-hoc* analyses showed that the VAAI-scores of all DHI-subgroups significantly differed from each other *(p* < 0.001 for all pairwise analyses).

Both healthy adults and participants with vestibular disorders showed significant differences in VAAI scores between the low, moderate, and high balance confidence groups (ABC subscales) indicating that fear avoidance beliefs and balance confidence are associated (Figure 2D). In the patient group, the *post-hoc* analyses revealed a significant lower VAAI score in the high balance confidence group [18.0 (11.1)] compared to the moderate [28.3 (9.8), *p* < 0.001] and low [33.4 (10.5), *p* < 0.001] balance confidence groups.

## 4 Discussion

### 4.1 Summary of the results

#### 4.1.1 Baseline characteristics across groups

Compared to healthy adults, participants with vestibular disorders presented with higher levels of perceived handicap, anxiety and depression scores, fear avoidance beliefs and lower confidence levels during balance activities. Indeed, a significantly higher prevalence of abnormal anxiety and depression scores (≥8/21 on the subscale scores of the HADS) was observed in participants with vestibular disorders (43 and 31.5%, respectively) compared to healthy adults (16.6% and 2.6%, respectively). These findings are consistent with previous research that found a higher psychological burden in persons with vestibular disorders compared to healthy adults (11–17). In addition, differences between the Dutch- and English-speaking participants with vestibular disorders were found, meaning that the population of Dutch-speaking participants with vestibular disorders showed a more severe perceived handicap and greater fear avoidance beliefs. The majority of the Dutch-speaking participants with a vestibular disorder were diagnosed with peripheral vestibular disorders other than BPPV (63.5%) whereas the most common diagnosis in the English-speaking participants with vestibular disorders was unspecified (37.9%). This might explain the worse scores in the Dutch-speaking participants with vestibular disorders. Participants with vestibular disorders with an unspecified diagnosis (*n* = 161) presented with significantly better scores on the VAAI (23.0 ± 12.9) and DHI (34.6 ± 20.9) compared to peripheral diagnoses other than BPPV (*n* = 218) [26.9 ± 14.2 on the VAAI (*p* = 0.006) and 42.8 ± 22.8 on the DHI (*p* = 0.001)]. However, until now, no consensus has been reached in the literature as to whether the severity of psychological factors differs between different types of vestibular disorders (18, 19). More research to further elaborate on this is recommended. Furthermore, it is worth noting that the English-speaking participants with vestibular disorders were recruited from both outpatient vestibular rehabilitation clinics and a tertiary care balance disorder clinic. In contrast, the Dutch-speaking participants with vestibular disorders were only recruited during consultations at specialized tertiary care centers. It is plausible that the latter group consisted of more complex diagnoses requiring tertiary care, thereby leading to a higher psychological burden. The Dutch-speaking participants with vestibular disorders reported a longer symptom duration [median of 12 months with an Inter Quartile Range (IQR) of 5–36] and consisted of more participants with chronic symptoms (87.7%) compared to the English-speaking (median of 8 months with an IQR of 3–24 and chronic symptoms in 79.2% of the participants), which could have contributed to a higher perceived handicap and fear avoidance beliefs as well.

#### 4.1.2 Convergent validity of VAAI

Significant associations were observed between the VAAI and all other questionnaires (DHI, ABC and HADS) in both healthy adults and participants with vestibular disorders. The strongest associations were present in participants with vestibular disorders. In summary, our results confirm previous research which identified the VAAI as a valid outcome measure in persons with balance and vestibular disorders (27).

#### 4.1.3 VAAI scores in HADS, DHI and ABC subgroups

Greater fear avoidance beliefs were observed in participants with elevated levels of anxiety, depression, perceived handicap and low levels of confidence during balance activities. Based on the internal fake-news-model of Hilber (10), fear avoidance beliefs might develop to avoid the sustained disturbed vestibular input and it's negative consequences. As fear avoidance beliefs eventually lead to activity limitations (40), presence of these beliefs should be recognized in a timely manner. Participants with vestibular disorders are less physically active compared to healthy adults (41), however, currently there are no studies available to our knowledge in which physical activity levels are compared between participants with vestibular disorders with or without the presence of fear avoidance beliefs. Therefore, future research elaborating on the relationship between fear avoidance beliefs and physical activity levels is recommended.

### 4.2 Clinical implications

Our results showed worse scores on all psychological outcome measures among participants with vestibular disorders compared to healthy adults. Assessing psychological factors in this population is necessary to consider appropriate treatment options such as relaxation techniques, CBT, and/or serotonergic medication (25, 26, 42, 43). In earlier studies, CBT resulted in desirable effects on dizziness, psychological factors, and balance performance (25, 26, 42). Furthermore, as literature reveals that persons with vestibular disorders who experience fear avoidance beliefs, might be less physically active (40), treatment options to boost physical activity should be explored. Enhancing physical activity in this population seems important, as higher physical activity levels are associated with better postural stability (44) and lower dizziness severity (45). Therefore, persons with vestibular disorders might benefit from monitoring physical activity by wearable sensors as this leads to an increase in daily step count and moderate to vigorous physical activity levels (46).

### 4.3 Limitations

In this study, multiple limitations were present. Although a large sample size was reached, the group of participants with vestibular disorders was quite heterogeneous with various vestibular diagnoses and a substantial percentage with unspecified diagnoses (30.4%). Moreover, data from the ABC was missing in the English-speaking participants with vestibular disorders, leading to an incomplete overview of psychological factors in participants with vestibular disorders across cultures. Regarding outcome measures, only patient reported outcome measures were used without presence of objective outcome measures such as vestibular function or balance performance tests. Earlier research led to conflicting evidence regarding the association between patient reported outcome measures and objective measurements (31, 47). Hence, investigating the association between the VAAI and objective measurements such as balance or vestibular function measures might lead to more consensus. Finally, the correlation coefficients indicate a strong relationship between the VAAI and the DHI but are likely inflated due to the VAAI including four items from the DHI (44.4% of the VAAI items). Two strengths of this study are the large sample size involving multiple sites in different countries and the fact that it provides normative data for multiple questionnaires from two countries and multiple sites. Additionally, this study provides information on the psychometric properties of the nine item version of the VAAI.

## 5 Conclusions

This study confirmed a higher psychological burden in persons with vestibular disorders compared to healthy adults. The higher psychological burden in persons with vestibular disorders supports the importance of the assessment of psychological factors in this population so that appropriate interventions can be provided. Additionally, the VAAI is a valid outcome measure for persons living with vestibular disorders across cultures.

## Data availability statement

The data is property of the University of Pittsburgh, the Erasmus Medical Centre, the Sint-Augustinus Hospital and University of Antwerp. Requests to access these datasets should be directed to LVa (lien.vanlaer@uantwerpen.be) who will then ask permission to all participating centers.

## Ethics statement

This multicenter study was approved by the University of Antwerp Ethics Committee (reference numbers: 18/12/162 and 21/12/181) and the UMC Erasmus Hospital Ethics Committee (reference number: WT/aj/MEC-2018-1190). All participants provided informed consent. The studies were conducted in accordance with the local legislation and institutional requirements. The participants provided their written informed consent to participate in this study.

## Author contributions

LVa: Data curation, Formal analysis, Investigation, Methodology, Visualization, Writing—original draft, Writing—review & editing. PD: Conceptualization, Data curation, Formal analysis, Investigation, Methodology, Supervision, Visualization, Writing—original draft, Writing—review & editing. LVe: Conceptualization, Data curation, Investigation, Methodology, Supervision, Writing—review & editing. EH: Conceptualization, Data curation, Investigation, Writing—review & editing. MS: Data curation, Investigation, Writing—review & editing. SW: Conceptualization, Data curation, Investigation, Methodology, Supervision, Writing—review & editing.
